# Epstein–Barr Virus-Specific Immune Control by Innate Lymphocytes

**DOI:** 10.3389/fimmu.2017.01658

**Published:** 2017-11-24

**Authors:** Christian Münz

**Affiliations:** ^1^Viral Immunobiology, Institute of Experimental Immunology, University of Zürich, Zürich, Switzerland

**Keywords:** natural killer cells, natural killer T cells, Vγ9Vδ2 T cells, lytic replication, infectious mononucleosis, NKG2D, CD27/CD70

## Abstract

Epstein–Barr virus (EBV) is a potent B cell transforming pathogen in humans. In most persistently EBV-infected individuals, potent cytotoxic lymphocyte responses prevent EBV-associated pathologies. In addition to comprehensive adaptive T cell responses, several innate lymphocyte populations seem to target different stages of EBV infection and are compromised in primary immunodeficiencies that render individuals susceptible to symptomatic EBV infection. In this mini-review, I will highlight the functions of natural killer, γδ T cells, and natural killer T cells during innate immune responses to EBV. These innate lymphocyte populations seem to restrict both lytic replication and transforming latent EBV antigen expression. The mechanisms underlying the recognition of these different EBV infection programs by the respective innate lymphocytes are just starting to become unraveled, but will provide immunotherapeutic strategies to target pathologies that are associated with the different EBV infection programs.

## Introduction on Innate Lymphocytes

Epstein–Barr virus (EBV) is a common human γ-herpesvirus that persistently infects more than 90% of the human adult population. At the same time, it was the first human candidate tumor virus that was discovered (1, 2) and remains to date the only human pathogen that can readily transform human B cells into immortal continuously growing lymphoblastoid cell lines (LCLs) (3). Even so EBV contributes with 1–2% to the overall tumor burden in humans (4), the majority of infected individuals carry EBV for life without symptoms. This peaceful coexistence is thought to be maintained by cytotoxic lymphocytes, which massively expand during symptomatic primary EBV infection, called infectious mononucleosis (IM), can be used to treat some EBV-associated malignancies and are affected by primary or secondary immunodeficiencies that predispose for EBV-driven pathologies, such as human immunodeficiency virus-associated lymphomas (5–7).

Among these cytotoxic lymphocytes, adaptive CD8^+^ T cell responses to EBV have been best characterized and single peptide epitope specificities against early lytic EBV antigens constitute in some individuals up to 40% of the massively expanded CD8^+^ T cell compartment during IM (8). Much less is known about innate cytotoxic lymphocyte compartments during EBV infection, including natural killer (NK), natural killer T (NKT), and γδ T cells. Nevertheless, they can utilize the same eomesodermin-dependent perforin, granzymes, and death receptor ligands to eliminate EBV-infected cells by cytotoxicity (9). Furthermore, they exist at much higher frequencies than individual CD8^+^ T cell clones at sites of primary EBV infection, like tonsils (more than 10^10^ more frequent), and therefore can more rapidly respond to pathogen encounter, ensuring the survival of the infected individual until specific T cells have been clonally expanded. However, their target cell recognition is not directed against EBV protein-derived peptides presented on major histocompatibility complex (MHC) molecules, but instead they recognize infected targets with their germ line encoded receptors or invariant T cell receptors. Activation of innate lymphocytes depend on loss of MHC class I molecules from the surface, stress induced ligand upregulation, glycolipid presentation on non-classical MHC class I molecules, or mevalonate metabolite recognition in the context of butyrophilin (BTN) family members (10–14). As I will discuss below, these different target recognition mechanisms seem to be used to target different stages of EBV infection, thereby achieving a similarly comprehensive immune control over all EBV infection programs as T cells that target antigens of the different EBV life cycles. Thereby, primary immunodeficiencies that affect NK, NKT, or γδ T cells might manifest with different EBV-associated pathologies. A better understanding of which EBV pathology might be targeted by which innate lymphocyte compartment might enable us to utilize these innate cytotoxic lymphocytes in addition to classical T cells for respective immunotherapies.

## NK Cells in the Prevention of Symptomatic Primary EBV Infection

Natural killer cells are the preeminent cytotoxic innate lymphocytes, which have been originally described for their spontaneous cytotoxicity against infected and tumor targets (15–17). In particular, deficiencies in NK cells predispose in humans for herpesvirus-driven pathologies (18). It was indeed described early on that NK cells also expand during IM (19–22). IM symptoms are thought to be caused by the associated lymphocytosis of CD8^+^ T cells, which primarily recognize lytic EBV antigens that are expressed during infectious virus production (23). Indeed, NK cells also preferentially recognize lytically EBV-replicating cells (22, 24, 25). Depletion of NK cells in mice with reconstituted human immune system components (HIS mice) increases viral loads and CD8^+^ T cell lymphocytosis only for wild-type (wt), but not lytic EBV replication incompetent BZLF1-deficient EBV (25). The respective NK cell-depleted and wt EBV-infected HIS mice also develop more EBV-associated B cell lymphomas and need to be sacrificed due to weight loss 6 weeks after infection (25). HIS mice share an early differentiated NK cell compartment with newborns and young children (26). The respective NKG2A^+^killer immunoglobulin-like receptor (KIR)^−^ NK cells preferentially expand during IM and recognize lytically EBV-replicating cells (21, 22). Interestingly, these early differentiated NK cells are continuously lost during the first decade of life and get successively replaced by KIR^+^ NK cell accumulation (22, 27). This coincides with an increased risk to develop IM when primary infection is delayed into adolescence (5). Recognition of lytic EBV replication might be mediated by the downregulation of MHC class I molecules and upregulation of NKG2D and DNAM-1 ligands on lytically EBV-replicating B cells (24, 28), tilting the balance of inhibitory, and activating NK cell receptor signaling toward activation. In contrast, EBV transformed B cells with the expression of all latent EBV antigens (LCLs) are only efficiently recognized by NK cells in the allogeneic MHC class I mismatched setting. This allows the recruitment of KIR^+^ NK cells to the response and can be harnessed in mixed MHC class I mismatched human immune system reconstitution from two hematopoietic progenitor cell donors in HIS mice (29). Although NK cells in these mixed reconstituted HIS mice have a decreased cytotoxicity against MHC class I negative target cells and are therefore less licensed, they control EBV infection better by NK cells (29). This results presumably from NK cell recognition of the MHC class I mismatched EBV-infected B cells, recruiting KIR^+^ NK cells to the innate immune response to EBV. Such allorecognition is currently being harnessed for NK cell-dependent immunotherapies of acute myeloid leukemias (30), but could also be harnessed against persistent infections that reactivate during bone marrow transplantation and home to the hematopoietic lineage. Thus, NK cells preferentially target lytic EBV replication, but might be therapeutically beneficial to target also other stages of EBV infection in the allogeneic setting.

## γδ T Cells and Their Restriction of EBV Latency

Natural killer cells are by far not the only cytotoxic innate lymphocytes that react to EBV infection. In a subset of EBV-positive children (25–50%), Vγ9Vδ2 T cells are also expanded (31). These human T cells with an invariant γδ T cell receptor do not exist in mice and recognize pyrophosphate-containing molecules that are generated in the mevalonate metabolism (32). Interestingly, Vγ9Vδ2 T cell recognition of these phosphoantigens (pAgs) depends on the BTN 3A1 molecule (CD277), but how BTN3A1 supports pAg recognition, remains unclear (32). In addition, γδ T cells can utilize the NK cell receptor NKG2D for target cell recognition (32), which has previously been described to be important in lytic EBV replication recognition by NK cells (24, 28). Interestingly, these Vγ9Vδ2 T cells seem to preferentially recognize EBV-infected B cell lines that express the nuclear antigen 1 of EBV (EBNA1) as the sole viral protein, so-called EBV latency I (31). This latency I is found in Burkitt’s lymphoma (BL), the most common childhood tumor in Sub-Saharan Africa and homeostatically proliferating EBV-infected memory B cells (33). Interestingly, such non-transformed EBV-infected memory B cells are thought to be the reservoir of EBV persistence (34), accumulate in the peripheral blood of IM patients (35), and might drive Vγ9Vδ2 T cell expansion in children, which sometimes have viral loads as high as IM patients (36). Indeed, BTN3A1 and NKG2D are required to expand Vγ9Vδ2 T cells with BL cell lines in donors who are susceptible for this expansion (31). Similarly, pAg stimulation of Vγ9Vδ2 T cells in HIS mice was able to prevent outgrowth of adoptively transferred EBV transformed LCLs *in vivo* (37). These activated Vγ9Vδ2 T cells also required their invariant T cell receptor and NKG2D for LCL recognition. In this study, Vγ9Vδ2 T cells seem to eliminate EBV transformed LCLs primarily by FasL- and TRAIL-mediated programmed cell death induction. Moreover, adoptive transfer of Vγ9Vδ2 T cells into HIS mice, in which EBV-associated lymphoma formation was induced by EBV infection, prevented tumorigenesis (38). Even 3 weeks after infection, adoptive transfer of activated Vγ9Vδ2 T cells was still able to reduce tumor burden substantially. These data suggest that Vγ9Vδ2 T cells preferentially expand to EBV latency I-infected B cells, but, once activated, can also target other EBV latencies, including latency III carrying EBV transformed LCLs. However, it remains unclear why this Vγ9Vδ2 T cell expansion can only be achieved in some donors and how pAg presentation or mevalonate metabolism is regulated during the different EBV latency programs. Nevertheless, Vγ9Vδ2 T cells seem to complement NK cells by recognizing latent EBV infection, while the latter innate lymphocyte subset preferentially controls lytic EBV replication. A combination of both cytotoxic innate lymphocyte subsets could be beneficial to target EBV infection.

## NKT Cell-Mediated Immune Control of EBV-Driven B Cell Transformation

Similar to our lack of understanding of how EBV regulates the mevalonate metabolism for Vγ9Vδ2 T cell recognition, also NKT cell recognition of EBV-infected B and epithelial cells is poorly understood, even so cytotoxicity of CD8^+^ NKT cells against EBV latency II Hodgkin lymphoma (HL) and nasopharyngeal carcinoma (NPC) cells was previously reported (39). NKT cells carry the invariant Vα24-Jα18/Vβ11 T cell receptor and recognize glycolipids that are presented on the non-classical MHC class I molecule CD1d (11). CD1d has been reported to be downregulated on fully EBV transformed LCLs (40). Nevertheless, EBV infection of primary human B cells and LCL outgrowth can be restricted by NKT cells, and restoring CD1d expression on LCLs allows NKT cells to recognize EBV latency III (40). These data suggest that during B cell infection and transformation CD1d ligands are produced and presented on CD1d that allow for NKT cell recognition. Therefore, NKT cells can also restrict EBV-induced tumorigenesis *in vivo* (39). In particular, CD8^+^ NKT cells can directly lyse EBV positive HL and NPC cells and produce IFN-γ, which augments protective Th1 responses against EBV infection (39). CD4^+^ NKT cells, which mainly produce IL-4 and bias immune responses toward Th2 polarization, do not seem to be able to control EBV on their own, but synergize with CD8^+^ NKT cells for improved immune control (39). While NKT cells are reduced in the peripheral blood of HL patients (39), they seem to be enriched in the tumor tissue (41). The HL and NPC associated EBV latency II with expression of three EBV latent antigens, namely EBNA1 and the two latent membrane proteins 1 and 2 (LMP1 and 2), can also be found in germinal center (GC) B cells of healthy EBV carriers (42). Therefore, NKT cells might play a role in restricting EBV latency II in GC B cells and epithelial cells. The latter might, however, only occur during NPC tumorigenesis, because EBV seems to mainly induce lytic replication in epithelial cells of healthy EBV carriers (43).

## Primary Immunodeficiencies That Compromise EBV-Specific Immune Control

The above discussed studies seem to indicate that several human innate lymphocyte subsets target different stages of EBV infection with NK cells recognizing lytic replication, Vγ9Vδ2 T cells reacting to EBV latency I and maybe III, and NKT cells providing restriction of EBV latency II. Can further evidence for this differential targeting of EBV by innate lymphocytes be gleaned from primary immunodeficiencies that predispose for EBV-associated pathologies (7, 44) and compromise these innate lymphocyte compartments?

The selective loss of NK, NKT, or γδ T cells is rare in primary immunodeficiencies. Usually, the respective mutations affect multiple immune compartments like the GATA2 mutation that was later characterized in the original patient with susceptibility to herpesvirus infections and decreased NK cell activity (18, 45). This mutation results in low numbers of B, CD4^+^ T, NK, dendritic, and monocytic cells. The associated uncontrolled EBV infection manifests in fulminant IM, hemophagocytic lymphohistiocytosis (HLH), and chronic active EBV (CAEBV). Similarly, mutations in the cytotoxic machinery (perforin, Munc13-4, and Munc18-2) that predispose for HLH and CAEBV affect all cytotoxic lymphocytes (46–48). Furthermore, the mutations in SLAM-associated protein (SAP) and X-linked inhibitor of apoptosis that result in X-linked lymphoproliferative diseases (XLP) 1 and 2 affect many lymphocytes and also result in fulminant IM and HLH (49–53), even so also NKT cell development is compromised in XLP1 patients (54, 55). Therefore, overall loss of cytotoxic lymphocyte control of EBV infection seems to result in uncontrolled IM, CAEBV, and HLH. However, other primary immunodeficiencies seem to be more selective, both with respect to clinical manifestation and loss of cytotoxic lymphocytes. In this regard, patients with mutations in IL-2 inducible T cell kinase (ITK) lack all NKT cells and present sometimes with HL (56–63). Similarly, CD70 deficiency predisposes for HL (64, 65), but so far only the deficiency of CD8^+^ T cells in recognizing EBV transformed B cells has been characterized. While in four of the five patients with CD70 deficiency no information about NKT cell numbers were given (64), in one patient NKT cell numbers were at least fivefold decreased. Thus, it is tempting to speculate that primary immunodeficiences, resulting from ITK and CD70 mutations, more prominently predispose for loss of NKT cell-mediated innate immune control and thereby favor uncontrolled EBV latency II, as in HL.

Even so CD70 is so far the only known ligand of CD27, CD27 mutations predispose for a much larger spectrum of EBV-associated pathologies, including HLH and different EBV-associated lymphomas, and also increase the mortality of affected individuals (66–68). It has been speculated that this results from ligand-independent signaling events of CD27 that are compromised in addition to T and NK cell recognition of LCLs (44). In addition to CD27, mutations in the magnesium transporter MagT1 and the transcription factor NFκB1 compromise NK cell recognition and predispose for EBV-induced lymphoproliferations and lymphomas (28, 69–74). These have been suggested to compromise NKG2D, TNF receptor (e.g., CD27), and SLAM receptor family (SAP dependent) signaling (28, 73). These receptors are crucial costimulatory molecules and activating receptors on CD8^+^ T and NK cells, respectively. More selective NK cell deficiencies have been reported for mutations in the minichromosome maintenance complex component 4 (MCM4) and the Fcγ receptor 3A (CD16) (75–78). Both types of mutations diminish or functionally impair the CD56^dim^CD16^+^ NK cell compartment, which contributes to the early differentiated NKG2A^+^KIR^−^ NK cells that were found to restrict lytic EBV replication (22, 25). In addition, they could mediate further restriction of lytic EBV replication by CD16-mediated antibody-dependent cellular cytotoxicity against late lytic viral glycoproteins. Patients with MCM4 and CD16 mutations present with EBV-induced lymphoproliferative diseases, including EBV-positive Castleman’s disease in the case of CD16 mutations. These selective NK cell deficiencies could point toward ill controlled lytic EBV infection that stimulates these lymphoproliferations.

In contrast to NKT and NK cells, Vγ9Vδ2 T cells have not received much attention in the characterization of primary immunodeficiencies that predispose for EBV pathologies. However, from the above described pathways that are affected by these, several are predicted to affect also Vγ9Vδ2 T cell function. Downstream of the TCR signaling, which in the case of Vγ9Vδ2 T cells engages pAgs in the context of BTN3A1, ITK phosphorylates PLCγ1, which elicits Ca^2+^ flux and phosphatidylinositol-4,5-bisphosphate cleavage to release diacylglycerol that in turn activates the guanine nucleotide exchange factor RasGRP1, whose mutations also predispose for EBV-associated B cell lymphomas (79). PLCγ1 activation is also Mg^2+^ dependent and thereby influenced by MagT1 function. Thus, mutations in ITK, MagT1, and RasGRP1 affect T cell receptor signaling and predispose for EBV-associated pathologies. Furthermore, NKG2D is a prominent coreceptor on Vγ9Vδ2 T cells and elicits NFκB1-dependent gene transcription (31). NKG2D and NFκB1 are affected by primary immunodeficiencies with EBV pathologies that result from mutations in the magnesium transporter MagT1 and the transcription factor NFκB1, respectively (28, 73). Finally, cytotoxicity of Vγ9Vδ2 T cells is also affected by the perforin, Munc13-4, and Munc18-2 mutations. These considerations suggest that T cell receptor signaling, costimulation, and effector functions of Vγ9Vδ2 T cells are compromised in some primary immunodeficiencies that predispose for EBV pathologies.

Apart from these immunodeficiencies whose genes can be related to innate lymphocyte function, other more general deficiencies like the mutations in the actin-binding protein coronin 1A and CTP synthase 1 are associated with NKT cell loss and EBV-associated lymphoproliferative diseases (80, 81). Furthermore, loss-of-function mutations in phosphatidylinositol-3-kinase subunit 110δ diminish NK cell killing and results in EBV viremia (82). Thus, primary immunodeficiences in the perforin machinery of cytotoxic lymphocytes, their costimulatory molecules, DNA-binding proteins that are required for their differentiation, and some less well-mechanistically understood gene products diminish innate lymphocyte activity and predispose for EBV-associated pathologies. These are summarized in Table 1.

**Table 1:** Primary immunodeficiencies that are associated with loss of immune control by innate lymphocytes and EBV-associated pathologies.

Affected protein	EBV-associated pathology	Affected innate lymphocytes	Reference
**Cytotoxic machinery**			
Perforin	CAEBV, HLH	NK, NKT, γδT	(46)
Munc13-4	CAEBV, HLH	NK, NKT, γδT	(47)
Munc18-2	CAEBV, HLH	NK, NKT, γδT	(48)
**DNA-binding proteins**			
GATA2	CAEBV, HLH	NK	(18, 45)
MCM4	EBV lymphoma	NK	(75, 76)
NF-κB1	EBV lymphoma	NK	(73, 74)
**Costimulatory receptors and their ligands**			
CD27	EBV lymphoma	NKT	(66–68)
CD70	EBV-positive Hodgkin’s lymphoma	NKT	(64, 65)
CD16	EBV-positive Castleman’s disease	NK	(77, 78)
NKG2D and TCR (because of MagT1 deficiency)	EBV lymphoma	NK, γδT	(69–72)
**Signaling molecules**			
SAP	EBV lymphoma, IM, HLH	NKT	(49–51, 54, 55)
ITK	EBV lymphoma	NKT	(56–63)
RasGRP1	EBV lymphoma	NKT	(79)
PI3K 110δ	EBV viremia	NK	(82)
**Others**			
XIAP	IM, HLH	NKT	(52, 53)
Coronin 1A	EBV lymphoma	NKT	(80)
CTP synthase 1	IM, EBV lymphoma	NKT	(81)

*CAEBV, chronic active EBV; HLH, hemophagocytic lymphohistiocytosis; IM, infectious mononucleosis; NK, natural killer; EBV, Epstein–Barr virus; NKT, natural killer T; SAP, SLAM-associated protein; XIAP, X-linked inhibitor of apoptosis; ITK, inducible T cell kinase; MCM4, minichromosome maintenance complex component 4; PI3K, phosphatidylinositol-3-kinase*.

## Conclusion and Outlook

**Figure 1:**
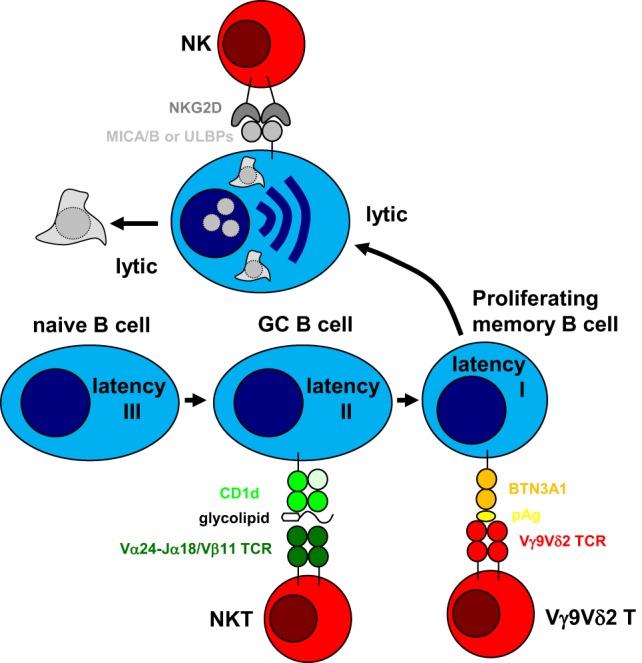
Innate lymphocytes target different stages of Epstein–Barr virus (EBV) infection. EBV was suggested to drive B cell differentiation by expressing all eight latent EBV proteins (latency III) in tonsillar naïve B cells and rescuing germinal center (GC) B cells with the expression of three latent EBV proteins (latency II) toward memory B cells. In homeostatically proliferating memory B cells, only one latent EBV protein is expressed for viral genome maintenance (latency I). From this infected memory B cell pool, EBV can reactivate into virus producing lytic replication, most likely after B cell receptor engagement. Natural killer (NK) cells have been shown to preferentially recognize lytic EBV replication and NKG2D has been suggested as an activating receptor involved in this recognition after upregulation of its MICA/B and ULBP ligands. In a subgroup of infected individuals, Vγ9Vδ2 T cells can be stimulated by EBV latency I Burkitt’s lymphoma cell lines and recognize these by mevalonate metabolite recognition in a butyrophilin (BTN) 3A1-dependent fashion. Finally, natural killer T (NKT) cells have been suggested to recognize EBV latency II in Hodgkin’s lymphoma cell lines, presumably by recognizing glycolipid presentation on CD1d. Thus, cytotoxic innate lymphocytes can target different stages of EBV infection.

The above outlined arguments suggest a division of labor among innate lymphocytes in targeting different programs of EBV infection. While NK cells might preferentially eliminate lytically EBV replicating cells, and immunodeficiencies that affect them could primary result in lymphoproliferations, NKT cells might be superior in restricting Hodgkin’s lymphoma and especially affected by ITK and CD70 deficiencies. Finally, Vγ9Vδ2 T cells might be able to target BL cells and LCLs. In combination, NK, NKT, and Vγ9Vδ2 T cells could therefore restrict EBV latencies I–III and lytic replication (Figure 1). This comprehensive immune control by innate lymphocytes might be especially important during early primary infection before protective CD8^+^ T cell responses have been primed. A better understanding of how these innate lymphocyte subsets collaborate during primary EBV infection could provide insights why IM preferentially develops in adolescence and which subgroup of these are especially at risk. Furthermore, characterizing how NK, NKT, and Vγ9Vδ2 T cells recognize EBV-infected cells and which infection programs in virus-associated malignancies are especially susceptible to this recognition could suggest immunotherapeutic approaches against the respective tumors, harnessing these innate lymphocytes.

## Author Contributions

The author confirms being the sole contributor of this work and approved it for publication.

## Conflict of Interest Statement

The author declares that the research was conducted in the absence of any commercial or financial relationships that could be construed as a potential conflict of interest.

## References

[1] Epstein MA Achong BG Barr YM Virus particles in cultured lymphoblasts from Burkitt’s lymphoma. Lancet (1964) 1:702–3. 10.1016/S0140-6736(64)91524-7 14107961

[2] Epstein MA Henle G Achong BG Barr YM Morphological and biological studies on a virus in cultured lymphoblasts from Burkitt’s lymphoma. J Exp Med (1964) 121:761–70. 10.1084/jem.121.5.761 PMC2138004 14278230

[3] Cesarman E. Gammaherpesviruses and lymphoproliferative disorders. Annu Rev Pathol (2014) 9:349–72. 10.1146/annurev-pathol-012513-104656 24111911

[4] Cohen JI Fauci AS Varmus H Nabel GJ. Epstein-Barr virus: an important vaccine target for cancer prevention. Sci Transl Med (2011) 3(107):107fs7. 10.1126/scitranslmed.3002878 22049067 PMC3501269

[5] Luzuriaga K Sullivan JL Infectious mononucleosis. N Engl J Med (2010) 362(21):1993–2000. 10.1056/NEJMcp1001116 20505178

[6] Gottschalk S Rooney CM Heslop HE. Post-transplant lymphoproliferative disorders. Annu Rev Med (2005) 56:29–44. 10.1146/annurev.med.56.082103.104727 15660500

[7] Cohen JI. Primary immunodeficiencies associated with EBV disease. Curr Top Microbiol Immunol (2015) 390(Pt 1):241–65. 10.1007/978-3-319-22822-8_10 26424649 PMC6349415

[8] Callan MF Tan L Annels N Ogg GS Wilson JD O’Callaghan CA Direct visualization of antigen-specific CD8^+^ T cells during the primary immune response to Epstein-Barr virus In vivo. J Exp Med (1998) 187(9):1395–402. 10.1084/jem.187.9.1395 9565632 PMC2212279

[9] Russell JH Ley TJ. Lymphocyte-mediated cytotoxicity. Annu Rev Immunol (2002) 20:323–70. 10.1146/annurev.immunol.20.100201.131730 11861606

[10] Vivier E Raulet DH Moretta A Caligiuri MA Zitvogel L Lanier LL Innate or adaptive immunity? The example of natural killer cells. Science (2011) 331(6013):44–9. 10.1126/science.1198687 21212348 PMC3089969

[11] Chandra S Kronenberg M. Activation and function of iNKT and MAIT cells. Adv Immunol (2015) 127:145–201. 10.1016/bs.ai.2015.03.003 26073984

[12] Chien YH Meyer C Bonneville M Gammadelta T cells: first line of defense and beyond. Annu Rev Immunol (2014) 32:121–55. 10.1146/annurev-immunol-032713-120216 24387714

[13] Ljunggren HG Karre K. Host resistance directed selectively against H-2-deficient lymphoma variants. Analysis of the mechanism. J Exp Med (1985) 162(6):1745–59. 10.1084/jem.162.6.1745 3877776 PMC2187973

[14] Correa I Corral L Raulet DH. Multiple natural killer cell-activating signals are inhibited by major histocompatibility complex class I expression in target cells. Eur J Immunol (1994) 24(6):1323–31. 10.1002/eji.1830240613 8206092

[15] Herberman RB Nunn ME Lavrin DH. Natural cytotoxic reactivity of mouse lymphoid cells against syngeneic acid allogeneic tumors. I. Distribution of reactivity and specificity. Int J Cancer (1975) 16(2):216–29. 10.1002/ijc.2910160204 50294

[16] Kiessling R Klein E Wigzell H “Natural” killer cells in the mouse. I. Cytotoxic cells with specificity for mouse Moloney leukemia cells. Specificity and distribution according to genotype. Eur J Immunol (1975) 5(2):112–7. 10.1002/eji.1830050208 1234049

[17] Trinchieri G Santoli D. Anti-viral activity induced by culturing lymphocytes with tumor-derived or virus-transformed cells. Enhancement of human natural killer cell activity by interferon and antagonistic inhibition of susceptibility of target cells to lysis. J Exp Med (1978) 147(5):1314–33. 10.1084/jem.147.5.1314 650156 PMC2184280

[18] Biron CA Byron KS Sullivan JL Severe herpesvirus infections in an adolescent without natural killer cells. N Engl J Med (1989) 320(26):1731–5. 10.1056/NEJM198906293202605 2543925

[19] Williams H McAulay K Macsween KF Gallacher NJ Higgins CD Harrison N The immune response to primary EBV infection: a role for natural killer cells. Br J Haematol (2005) 129(2):266–74. 10.1111/j.1365-2141.2005.05452.x 15813855

[20] Dunmire SK Grimm JM Schmeling DO Balfour HH Jr Hogquist KA. The incubation period of primary Epstein-Barr virus infection: viral dynamics and immunologic events. PLoS Pathog (2015) 11(12):e1005286. 10.1371/journal.ppat.1005286 26624012 PMC4666617

[21] Hendricks DW Balfour HH Jr Dunmire SK Schmeling DO Hogquist KA Lanier LL Cutting edge: NKG2C^hi^CD57^+^ NK cells respond specifically to acute infection with cytomegalovirus and not Epstein-Barr virus. J Immunol (2014) 192(10):4492–6. 10.4049/jimmunol.1303211 24740502 PMC4013527

[22] Azzi T Lunemann A Murer A Ueda S Beziat V Malmberg KJ Role for early-differentiated natural killer cells in infectious mononucleosis. Blood (2014) 124(16):2533–43. 10.1182/blood-2014-01-553024 25205117 PMC4199955

[23] Taylor GS Long HM Brooks JM Rickinson AB Hislop AD. The immunology of Epstein-Barr virus-induced disease. Annu Rev Immunol (2015) 33:787–821. 10.1146/annurev-immunol-032414-112326 25706097

[24] Pappworth IY Wang EC Rowe M. The switch from latent to productive infection in Epstein-Barr virus-infected B cells is associated with sensitization to NK cell killing. J Virol (2007) 81(2):474–82. 10.1128/JVI.01777-06 17079298 PMC1797427

[25] Chijioke O Muller A Feederle R Barros MH Krieg C Emmel V Human natural killer cells prevent infectious mononucleosis features by targeting lytic Epstein-Barr virus infection. Cell Rep (2013) 5(6):1489–98. 10.1016/j.celrep.2013.11.041 24360958 PMC3895765

[26] Strowig T Chijioke O Carrega P Arrey F Meixlsperger S Ramer PC Human NK cells of mice with reconstituted human immune system components require preactivation to acquire functional competence. Blood (2010) 116(20):4158–67. 10.1182/blood-2010-02-270678 20671122 PMC2993621

[27] Sundstrom Y Nilsson C Lilja G Karre K Troye-Blomberg M Berg L The expression of human natural killer cell receptors in early life. Scand J Immunol (2007) 66(2–3):335–44. 10.1111/j.1365-3083.2007.01980.x 17635811

[28] Chaigne-Delalande B Li FY O’Connor GM Lukacs MJ Jiang P Zheng L Mg2^+^ regulates cytotoxic functions of NK and CD8 T cells in chronic EBV infection through NKG2D. Science (2013) 341(6142):186–91. 10.1126/science.1240094 23846901 PMC3894782

[29] Landtwing V Raykova A Pezzino G Beziat V Marcenaro E Graf C Cognate HLA absence in trans diminishes human NK cell education. J Clin Invest (2016) 126(10):3772–82. 10.1172/JCI86923 27571408 PMC5096830

[30] Velardi A Ruggeri L Mancusi A. Killer-cell immunoglobulin-like receptors reactivity and outcome of stem cell transplant. Curr Opin Hematol (2012) 19(4):319–23. 10.1097/MOH.0b013e32835423c3 22555394

[31] Djaoud Z Guethlein LA Horowitz A Azzi T Nemat-Gorgani N Olive D Two alternate strategies for innate immunity to Epstein-Barr virus: one using NK cells and the other NK cells and gammadelta T cells. J Exp Med (2017) 214(6):1827–41. 10.1084/jem.20161017 28468758 PMC5460997

[32] Chitadze G Oberg HH Wesch D Kabelitz D The ambiguous role of gammadelta T lymphocytes in antitumor immunity. Trends Immunol (2017) 38(9):668–78. 10.1016/j.it.2017.06.004 28709825

[33] Hochberg D Middeldorp JM Catalina M Sullivan JL Luzuriaga K Thorley-Lawson DA. Demonstration of the Burkitt’s lymphoma Epstein-Barr virus phenotype in dividing latently infected memory cells in vivo. Proc Natl Acad Sci U S A (2004) 101(1):239–44. 10.1073/pnas.2237267100 14688409 PMC314169

[34] Babcock GJ Decker LL Volk M Thorley-Lawson DA EBV persistence in memory B cells in vivo. Immunity (1998) 9(3):395–404. 10.1016/S1074-7613(00)80622-6 9768759

[35] Babcock GJ Decker LL Freeman RB Thorley-Lawson DA. Epstein-Barr virus-infected resting memory B cells, not proliferating lymphoblasts, accumulate in the peripheral blood of immunosuppressed patients. J Exp Med (1999) 190(4):567–76. 10.1084/jem.190.4.567 10449527 PMC2195601

[36] Jayasooriya S de Silva TI Njie-jobe J Sanyang C Leese AM Bell AI Early virological and immunological events in asymptomatic Epstein-Barr virus infection in African children. PLoS Pathog (2015) 11(3):e1004746. 10.1371/journal.ppat.1004746 25816224 PMC4376400

[37] Xiang Z Liu Y Zheng J Liu M Lv A Gao Y Targeted activation of human Vgamma9Vdelta2-T cells controls Epstein-Barr virus-induced B cell lymphoproliferative disease. Cancer Cell (2014) 26(4):565–76. 10.1016/j.ccr.2014.07.026 25220446

[38] Zumwalde NA Sharma A Xu X Ma S Schneider CL Romero-Masters JC Adoptively transferred Vgamma9Vdelta2 T cells show potent antitumor effects in a preclinical B cell lymphomagenesis model. JCI Insight (2017) 2(13):93179 10.1172/jci.insight.93179 28679955 PMC5499361

[39] Yuling H Ruijing X Li L Xiang J Rui Z Yujuan W EBV-induced human CD8^+^ NKT cells suppress tumorigenesis by EBV-associated malignancies. Cancer Res (2009) 69(20):7935–44. 10.1158/0008-5472.CAN-09-0828 19808969

[40] Chung BK Tsai K Allan LL Zheng DJ Nie JC Biggs CM Innate immune control of EBV-infected B cells by invariant natural killer T cells. Blood (2013) 122(15):2600–8. 10.1182/blood-2013-01-480665 23974196

[41] Hudnall SD Betancourt E Barnhart E Patel J. Comparative flow immunophenotypic features of the inflammatory infiltrates of Hodgkin lymphoma and lymphoid hyperplasia. Cytometry B Clin Cytom (2008) 74(1):1–8. 10.1002/cyto.b.20376 18061945

[42] Babcock JG Hochberg D Thorley-Lawson AD. The expression pattern of Epstein-Barr virus latent genes in vivo is dependent upon the differentiation stage of the infected B cell. Immunity (2000) 13(4):497–506. 10.1016/S1074-7613(00)00049-2 11070168

[43] Hutt-Fletcher LM The long and complicated relationship between Epstein-Barr virus and epithelial cells. J Virol (2017) 91(1):e01677–16. 10.1128/JVI.01677-16 27795426 PMC5165189

[44] Tangye SG Palendira U Edwards ES. Human immunity against EBV-lessons from the clinic. J Exp Med (2017) 214(2):269–83. 10.1084/jem.20161846 28108590 PMC5294862

[45] Mace EM Hsu AP Monaco-Shawver L Makedonas G Rosen JB Dropulic L Mutations in GATA2 cause human NK cell deficiency with specific loss of the CD56^bright^ subset. Blood (2013) 121(14):2669–77. 10.1182/blood-2012-09-453969 23365458 PMC3617632

[46] Katano H Ali MA Patera AC Catalfamo M Jaffe ES Kimura H Chronic active Epstein-Barr virus infection associated with mutations in perforin that impair its maturation. Blood (2004) 103(4):1244–52. 10.1182/blood-2003-06-2171 14576041

[47] Rohr J Beutel K Maul-Pavicic A Vraetz T Thiel J Warnatz K Atypical familial hemophagocytic lymphohistiocytosis due to mutations in UNC13D and STXBP2 overlaps with primary immunodeficiency diseases. Haematologica (2010) 95(12):2080–7. 10.3324/haematol.2010.029389 20823128 PMC2995566

[48] Cohen JI Niemela JE Stoddard JL Pittaluga S Heslop H Jaffe ES Late-onset severe chronic active EBV in a patient for five years with mutations in STXBP2 (MUNC18-2) and PRF1 (perforin 1). J Clin Immunol (2015) 35(5):445–8. 10.1007/s10875-015-0168-y 25947952 PMC4504756

[49] Coffey AJ Brooksbank RA Brandau O Oohashi T Howell GR Bye JM Host response to EBV infection in X-linked lymphoproliferative disease results from mutations in an SH2-domain encoding gene. Nat Genet (1998) 20(2):129–35. 10.1038/2424 9771704

[50] Nichols KE Harkin DP Levitz S Krainer M Kolquist KA Genovese C Inactivating mutations in an SH2 domain-encoding gene in X-linked lymphoproliferative syndrome. Proc Natl Acad Sci U S A (1998) 95(23):13765–70. 10.1073/pnas.95.23.13765 9811875 PMC24894

[51] Sayos J Wu C Morra M Wang N Zhang X Allen D The X-linked lymphoproliferative-disease gene product SAP regulates signals induced through the co-receptor SLAM. Nature (1998) 395(6701):462–9. 10.1038/26683 9774102

[52] Rigaud S Fondaneche MC Lambert N Pasquier B Mateo V Soulas P XIAP deficiency in humans causes an X-linked lymphoproliferative syndrome. Nature (2006) 444(7115):110–4. 10.1038/nature05257 17080092

[53] Speckmann C Lehmberg K Albert MH Damgaard RB Fritsch M Gyrd-Hansen M X-linked inhibitor of apoptosis (XIAP) deficiency: the spectrum of presenting manifestations beyond hemophagocytic lymphohistiocytosis. Clin Immunol (2013) 149(1):133–41. 10.1016/j.clim.2013.07.004 23973892

[54] Nichols KE Hom J Gong SY Ganguly A Ma CS Cannons JL Regulation of NKT cell development by SAP, the protein defective in XLP. Nat Med (2005) 11(3):340–5. 10.1038/nm1189 15711562 PMC10655637

[55] Pasquier B Yin L Fondaneche MC Relouzat F Bloch-Queyrat C Lambert N Defective NKT cell development in mice and humans lacking the adapter SAP, the X-linked lymphoproliferative syndrome gene product. J Exp Med (2005) 201(5):695–701. 10.1084/jem.20042432 15738056 PMC2212840

[56] Huck K Feyen O Niehues T Ruschendorf F Hubner N Laws HJ Girls homozygous for an IL-2-inducible T cell kinase mutation that leads to protein deficiency develop fatal EBV-associated lymphoproliferation. J Clin Invest (2009) 119(5):1350–8. 10.1172/JCI37901 19425169 PMC2673872

[57] Linka RM Risse SL Bienemann K Werner M Linka Y Krux F Loss-of-function mutations within the IL-2 inducible kinase ITK in patients with EBV-associated lymphoproliferative diseases. Leukemia (2012) 26(5):963–71. 10.1038/leu.2011.371 22289921

[58] Stepensky P Weintraub M Yanir A Revel-Vilk S Krux F Huck K IL-2-inducible T-cell kinase deficiency: clinical presentation and therapeutic approach. Haematologica (2011) 96(3):472–6. 10.3324/haematol.2010.033910 21109689 PMC3046282

[59] Mansouri D Mahdaviani SA Khalilzadeh S Mohajerani SA Hasanzad M Sadr S IL-2-inducible T-cell kinase deficiency with pulmonary manifestations due to disseminated Epstein-Barr virus infection. Int Arch Allergy Immunol (2012) 158(4):418–22. 10.1159/000333472 22487848

[60] Ghosh S Bienemann K Boztug K Borkhardt A. Interleukin-2-inducible T-cell kinase (ITK) deficiency – clinical and molecular aspects. J Clin Immunol (2014) 34(8):892–9. 10.1007/s10875-014-0110-8 25339095 PMC4220104

[61] Bienemann K Borkhardt A Klapper W Oschlies I. High incidence of Epstein-Barr virus (EBV)-positive Hodgkin lymphoma and Hodgkin lymphoma-like B-cell lymphoproliferations with EBV latency profile 2 in children with interleukin-2-inducible T-cell kinase deficiency. Histopathology (2015) 67(5):607–16. 10.1111/his.12677 25728094

[62] Cipe FE Aydogmus C Serwas NK Tugcu D Demirkaya M Bicici FA ITK deficiency: how can EBV be treated before lymphoma? Pediatr Blood Cancer (2015) 62(12):2247–8. 10.1002/pbc.25648 26174447

[63] Cagdas D Erman B Hanoglu D Tavil B Kuskonmaz B Aydin B Course of IL-2-inducible T-cell kinase deficiency in a family: lymphomatoid granulomatosis, lymphoma and allogeneic bone marrow transplantation in one sibling; and death in the other. Bone Marrow Transplant (2017) 52(1):126–9. 10.1038/bmt.2016.185 27454071

[64] Abolhassani H Edwards ES Ikinciogullari A Jing H Borte S Buggert M Combined immunodeficiency and Epstein-Barr virus-induced B cell malignancy in humans with inherited CD70 deficiency. J Exp Med (2017) 214(1):91–106. 10.1084/jem.20160849 28011864 PMC5206499

[65] Izawa K Martin E Soudais C Bruneau J Boutboul D Rodriguez R Inherited CD70 deficiency in humans reveals a critical role for the CD70-CD27 pathway in immunity to Epstein-Barr virus infection. J Exp Med (2017) 214(1):73–89. 10.1084/jem.20160784 28011863 PMC5206497

[66] van Montfrans JM Hoepelman AI Otto S van Gijn M van de Corput L de Weger RA CD27 deficiency is associated with combined immunodeficiency and persistent symptomatic EBV viremia. J Allergy Clin Immunol (2012) 129(3):787–93.e6. 10.1016/j.jaci.2011.11.013 22197273 PMC3294016

[67] Salzer E Daschkey S Choo S Gombert M Santos-Valente E Ginzel S Combined immunodeficiency with life-threatening EBV-associated lymphoproliferative disorder in patients lacking functional CD27. Haematologica (2013) 98(3):473–8. 10.3324/haematol.2012.068791 22801960 PMC3659923

[68] Alkhairy OK Perez-Becker R Driessen GJ Abolhassani H van Montfrans J Borte S Novel mutations in TNFRSF7/CD27: clinical, immunologic, and genetic characterization of human CD27 deficiency. J Allergy Clin Immunol (2015) 136(3):703–12.e10. 10.1016/j.jaci.2015.02.022 25843314

[69] Li FY Chaigne-Delalande B Kanellopoulou C Davis JC Matthews HF Douek DC Second messenger role for Mg2^+^ revealed by human T-cell immunodeficiency. Nature (2011) 475(7357):471–6. 10.1038/nature10246 21796205 PMC3159560

[70] Dhalla F Murray S Sadler R Chaigne-Delalande B Sadaoka T Soilleux E Identification of a novel mutation in MAGT1 and progressive multifocal leucoencephalopathy in a 58-year-old man with XMEN disease. J Clin Immunol (2015) 35(2):112–8. 10.1007/s10875-014-0116-2 25504528 PMC6328310

[71] Patiroglu T Haluk Akar H Gilmour K Unal E Akif Ozdemir M Bibi S A case of XMEN syndrome presented with severe auto-immune disorders mimicking autoimmune lymphoproliferative disease. Clin Immunol (2015) 159(1):58–62. 10.1016/j.clim.2015.04.015 25956530

[72] Brigida I Chiriaco M Di Cesare S Cittaro D Di Matteo G Giannelli S Large deletion of MAGT1 gene in a patient with classic Kaposi sarcoma, CD4 lymphopenia, and EBV infection. J Clin Immunol (2017) 37(1):32–5. 10.1007/s10875-016-0341-y 27770395 PMC5226982

[73] Boztug H Hirschmugl T Holter W Lakatos K Kager L Trapin D NF-kappaB1 haploinsufficiency causing immunodeficiency and EBV-driven lymphoproliferation. J Clin Immunol (2016) 36(6):533–40. 10.1007/s10875-016-0306-1 27338827 PMC4940442

[74] Schipp C Nabhani S Bienemann K Simanovsky N Kfir-Erenfeld S Assayag-Asherie N Specific antibody deficiency and autoinflammatory disease extend the clinical and immunological spectrum of heterozygous NFKB1 loss-of-function mutations in humans. Haematologica (2016) 101(10):e392–6. 10.3324/haematol.2016.145136 27365489 PMC5046658

[75] Eidenschenk C Dunne J Jouanguy E Fourlinnie C Gineau L Bacq D A novel primary immunodeficiency with specific natural-killer cell deficiency maps to the centromeric region of chromosome 8. Am J Hum Genet (2006) 78(4):721–7. 10.1086/503269 16532402 PMC1424699

[76] Gineau L Cognet C Kara N Lach FP Dunne J Veturi U Partial MCM4 deficiency in patients with growth retardation, adrenal insufficiency, and natural killer cell deficiency. J Clin Invest (2012) 122(3):821–32. 10.1172/JCI61014 22354167 PMC3287233

[77] de Vries E Koene HR Vossen JM Gratama JW Borne AE Waaijer JL Identification of an unusual Fc gamma receptor IIIa (CD16) on natural killer cells in a patient with recurrent infections. Blood (1996) 88(8):3022–7. 8874200

[78] Grier JT Forbes LR Monaco-Shawver L Oshinsky J Atkinson TP Moody C Human immunodeficiency-causing mutation defines CD16 in spontaneous NK cell cytotoxicity. J Clin Invest (2012) 122(10):3769–80. 10.1172/JCI64837 23006327 PMC3461929

[79] Salzer E Cagdas D Hons M Mace EM Garncarz W Petronczki OY RASGRP1 deficiency causes immunodeficiency with impaired cytoskeletal dynamics. Nat Immunol (2016) 17(12):1352–60. 10.1038/ni.3575 27776107 PMC6400263

[80] Moshous D Martin E Carpentier W Lim A Callebaut I Canioni D Whole-exome sequencing identifies Coronin-1A deficiency in 3 siblings with immunodeficiency and EBV-associated B-cell lymphoproliferation. J Allergy Clin Immunol (2013) 131(6):1594–603. 10.1016/j.jaci.2013.01.042 23522482 PMC3824285

[81] Martin E Palmic N Sanquer S Lenoir C Hauck F Mongellaz C CTP synthase 1 deficiency in humans reveals its central role in lymphocyte proliferation. Nature (2014) 510(7504):288–92. 10.1038/nature13386 24870241 PMC6485470

[82] Kuehn HS Niemela JE Rangel-Santos A Zhang M Pittaluga S Stoddard JL Loss-of-function of the protein kinase C delta (PKCdelta) causes a B-cell lymphoproliferative syndrome in humans. Blood (2013) 121(16):3117–25. 10.1182/blood-2012-12-469544 23430113 PMC3630827

